# Machine–learning-enabled metasurface for direction of arrival estimation

**DOI:** 10.1515/nanoph-2021-0663

**Published:** 2022-01-11

**Authors:** Min Huang, Bin Zheng, Tong Cai, Xiaofeng Li, Jian Liu, Chao Qian, Hongsheng Chen

**Affiliations:** Interdisciplinary Center for Quantum Information, State Key Laboratory of Modern Optical Instrumentation, ZJU-Hangzhou Global Scientific and Technological Innovation Center, Zhejiang University, Hangzhou 310027, China; International Joint Innovation Center, Key Lab. of Advanced Micro/Nano Electronic Devices & Smart Systems of Zhejiang, The Electromagnetics Academy at Zhejiang University, Zhejiang University, Haining 314400, China; Jinhua Institute of Zhejiang University, Zhejiang University, Jinhua 321099, China; Air and Missile Defense College, Air Force Engineering University, Xi’an 710051, China

**Keywords:** direction of arrival estimation, metasurface, random forest

## Abstract

Metasurfaces, interacted with artificial intelligence, have now been motivating many contemporary research studies to revisit established fields, e.g., direction of arrival (DOA) estimation. Conventional DOA estimation techniques typically necessitate bulky-sized beam-scanning equipment for signal acquisition or complicated reconstruction algorithms for data postprocessing, making them ineffective for *in-situ* detection. In this article, we propose a machine-learning-enabled metasurface for DOA estimation. For certain incident signals, a tunable metasurface is controlled in sequence, generating a series of field intensities at the single receiving probe. The perceived data are subsequently processed by a pretrained random forest model to access the incident angle. As an illustrative example, we experimentally demonstrate a high-accuracy intelligent DOA estimation approach for a wide range of incident angles and achieve more than 95% accuracy with an error of less than 0.5° . The reported strategy opens a feasible route for intelligent DOA detection in full space and wide band. Moreover, it will provide breakthrough inspiration for traditional applications incorporating time-saving and equipment-simplified majorization.

## 1 Introduction

As a key technology in adaptive arrays, the direction of arrival (DOA) is widely used in mobile communication, radar, sonar, navigation, seismic detection, and medical imaging [1], [2], [3], [4], [5]. Realizing intelligent DOA detection has been a long-standing interesting topic, but it faces great challenges from the construction of tunable electromagnetic (EM) detector, machine-learning calculator, and complex EM environment interference. Metasurfaces retain a remarkable ability [6], [7], [8], [9] to manipulate the phases, amplitudes, and polarizations of EM waves and have been used to facilitate numerous applications, such as wireless communication systems [10], [11], [12], holographic imagers [13, 14], beam deflectors [15, 16], focusing devices [17], [18], [19], DOA estimations [20], [21], [22], [23], and other applications [24], [25], [26], [27]. Furthermore, tunable metasurfaces [28], [29], [30], [31], [32], [33], [34], [35], [36], realized by various means such as phase-change materials, mechanical regulation, and active devices (e.g., varactor diodes and PIN diodes), have also been exploited. Recently, with the emergence of artificial intelligence, the collaboration between tunable metasurfaces and machine learning has promoted applications in cloaks, imagers, holograms and beyond, providing intelligence and ease of use to metasurface-based devices [37], [38], [39], [40], [41], [42].

A traditional DOA estimation collects data through massive phased-array antennas driven by complicated feeding networks and requires considerable memory for reasoning. Researchers have attempted to simplify the physical equipment by replacing the phased-array antennas with a rotating antenna or (non-) uniform linear antenna arrays [43], [44], [45]. Understandably, the device was simplified, but the collection of a large amount of data is still unavoidable. Instead of focusing on the physical structure, analytical solution methods incorporating neural networks have been proposed to improve the computing performance [46], [47], [48]. Nevertheless, this approach remains limited by the traditional DOA detection principle. Considering a programmable metasurface as a physical random sampling device [20] or replacing a large number of antennas used to satisfy Nyquist’s theorem with coding metasurface [21], they can recover the DOA information by compressive sensing. Both approaches stem from the need to process large amounts of sample data. In addition, an eight-port antenna array and generalized regression neural network (GRNN) have been designed for simultaneous attainments of the DOA [22]. However, the GRNN relies on a massive original database to traverse an answer; in other words, it is not straightforward. Both approaches are always attached to large amounts of data. More directly, a direction-selective multichannel metasurface has been designed to obtain the DOA [23]. It is indicated with relatively complex requirements in actualization, where the spin-wave control is needed, and the interchannel insulation is an additional consideration. Overall, the current incoming wave detection still presents some challenges, such as harsh calculation conditions, complex physical equipment, and poor portability.

In this article, we introduce a metasurface-assisted DOA estimation method with machine learning to overcome such barriers. It uses a tunable metasurface for transmissive wave manipulation and adopts machine learning as an intelligent calculator for compactness and simplicity. This approach engages machine learning to focus directly on the relationship between the signals and DOA while not relying on signals from other channels, exempting it from large data processing challenges. The proposed method can achieve an accuracy of 95.6% with a $0.5^{{}^{\circ}}$ prediction error and can perform real-time incoming wave estimation in practice. Our metasurface-enabled DOA estimation approach provides some inspiration for uncomplicated, efficient, and time-saving intelligent DOA estimations in radar, wireless communication, and other fields.

## 2 Theoretical design

### 2.1 Principle of machine-learning-enabled metasurface for DOA estimation

**Figure 1:**
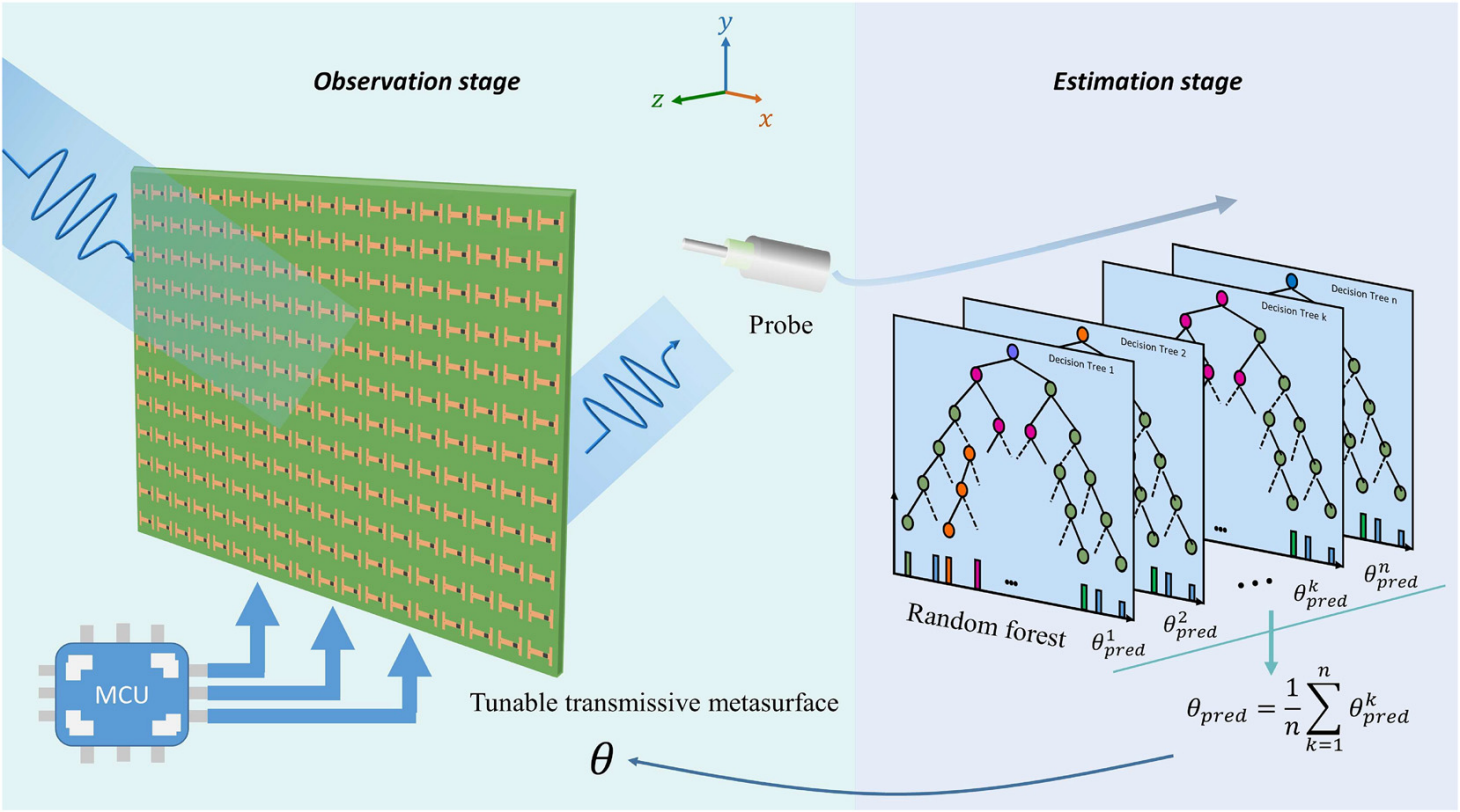
Schematic of machine-learning-enabled metasurface for DOA estimation. The metasurface-enabled DOA estimation method comprises a tunable transmissive metasurface and one single receiving probe in observation stage and random forect (RF) for prediction in estimation stage. The tunable metasurface, configured with a microcontrol unit, will switch to specific phase patterns multiple times to control the transmissive waves, as received by a single probe located in a fixed place. The amplitude data, containing adequate directional information in each phase pattern, will be delivered to the RF in real time.

The EM waves are considered to travel in the *z-direction*, and the metasurface is in the *xy-plane* (Figure 1). In spherical coordinates, the incident plane wave can be calculated as follows:

(1) $$P_{inc}=e^{ik\left(xsin\theta cos\varphi +ysin\theta sin\varphi \right)}$$

where k is the wave vector of the EM wave in free space, and (θ,φ) denotes the incident angles (azimuth angle φ , elevation angle θ ) of the source to be retrieved through the proposed DOA estimation technique. For one-dimensional DOA detection, i.e., $\varphi =0^{{}^{\circ}}$ , the incident signal can be rewritten as $P_{inc}=e^{ikxsin\theta }$ . By considering only the transmission field distribution of the detector located at $\vec{r}=\left(x_{t},y_{t},z_{t}\right)$ , the transmissive wave can be described as follows:

(2) $$P_{t}\left({\vec{r}}_{i}-\vec{r}\right)=\underset{i}{\sum }w_{i}\left({\vec{r}}_{i}-\vec{r}\right)\cdot t_{i}\left({\vec{r}}_{i}\right)\cdot P_{inc}^{i}\left({\vec{r}}_{i}\right)$$

Here, the transmission coefficient is $t=ae^{i\varphi }$ . Based on the Rayleigh–Sommerfeld diffraction [49], the diffraction coefficient is calculated as follows:

(3) $$w_{i}\left({\vec{r}}_{i}-\vec{r}\right)=\frac{z-z_{i}}{R^{2}\left(\frac{1}{2\pi R}-\frac{i}{\lambda }\right)\text{exp}\left(\frac{i2\pi R}{\lambda }\right)}$$

In the above, $R=\sqrt{{\left(x_{i}-x_{t}\right)}^{2}+{\left(y_{i}-y_{t}\right)}^{2}+{\left(z_{i}-z_{t}\right)}^{2}}$ . In this way, the EM waves transmitted through all multiple-phase modes carry the same incoming wave information. Machine learning suggests a possibility to extract the implicit relationship between the transmission waves and DOA.

**Figure 2:**
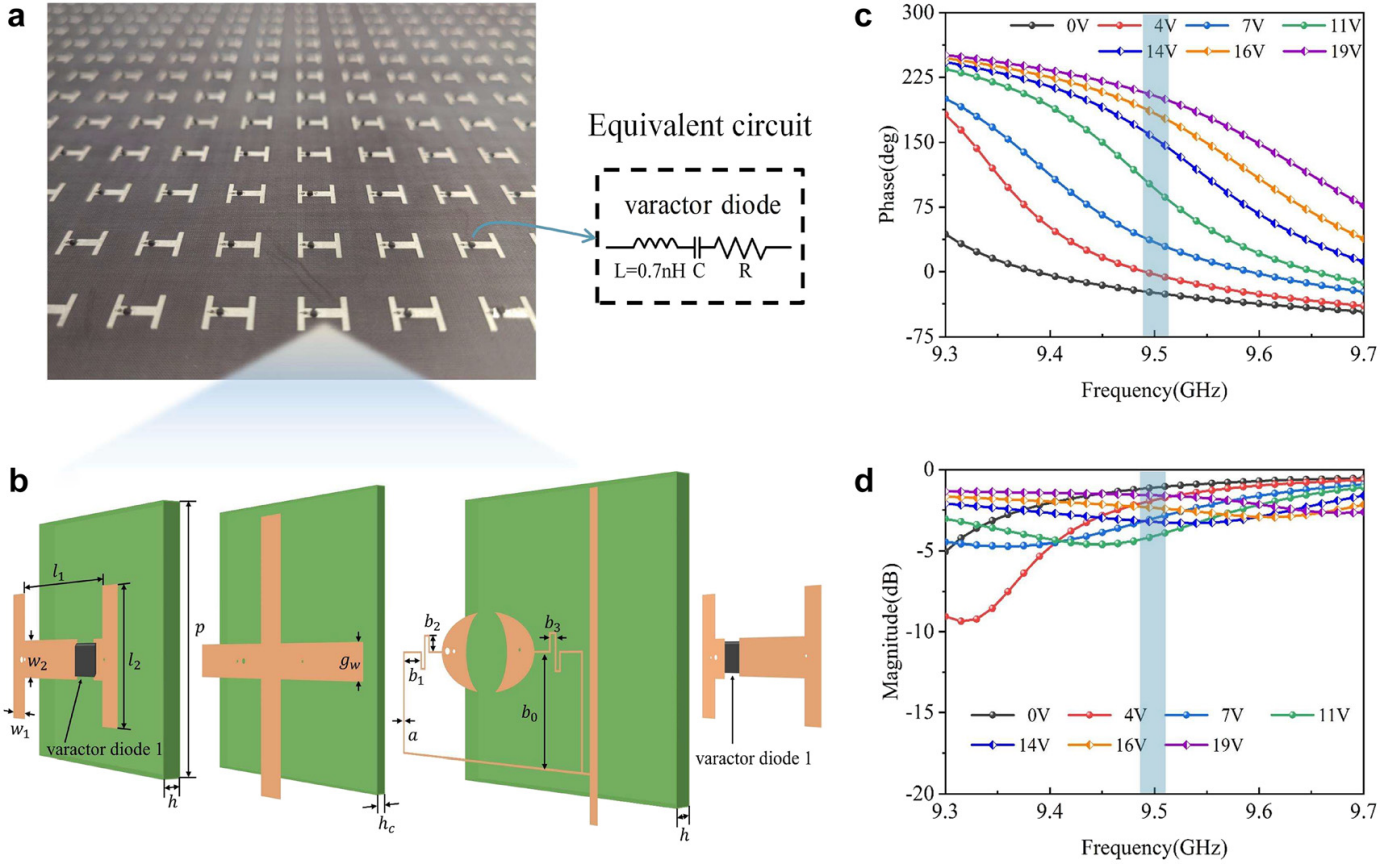
Design of the tunable transmissive metasurface. (a) Schematic of the proposed tunable transmissive metasurface. (b) Structure of the meta-atom. Incorporating two varactor diodes allows the phase of meta-atom to be controlled by supplying different bias voltages. The relationship between the bias voltage and capacitance is shown in the table (see in Supplementary Note 1). Additional detailed information about the structure is provided in Supplementary Note 1. (c) and(d) Phase and amplitude of the designed meta-atom. The colorful curves indicate that the varactor diode is controlled by different bias voltages.

The concept of metasurface-assisted DOA estimation method is illustrated in Figure 1. The entire stage is divided into observation and estimation stages. In the observation stage, a tunable metasurface generates a series of desired phase patterns by feeding different bias voltages. A single probe is located behind the metasurface to detect the transmissive waves. Notably, a horn antenna is also applicable for collecting data, but the probe is of low-cost, easy to process, and easy to integrate, which is more suitable for cost-effective DOA estimation. With the aid of machine learning, the estimation stage barely requires excessive data to derive and analyze the direction of the incoming waves every time. In the detection process, one only needs to call the trained model parameters directly, thereby reducing the memory consumption and improving the computational efficiency. As shown in Figure 2(a) and (b), the transmissive tunable metasurface consists of 40×20 meta-atoms, each with dimensions of $15\times 15\times 4.64{\text{ mm}}^{3}$ . Each meta-atom is integrated with two varactor diodes (SMV2019-079LF) for electronic control [24,50]. The two varactor diodes in each unit are connected in parallel (see Supplementary Note 1 for more detailed information). Moreover, the meta-atoms in the same column along the *y*-direction share the same bias voltage. Therefore, the metasurface can be treated as one-dimensional to manipulate the elevation angle θ . The transmitted waves can be arbitrarily modulated when passing through the tunable metasurface with diversified phase patterns.

Figure 2(c) and (d) show the phase and amplitude response of each meta-atom under different bias voltages to the varactor diodes, as calculated by the commercial software, CST Microwave Studio. It can be observed that when working at 9.5 GHz, the maximum phase coverage reaches over 235° . It is worth noting that although a 360-degree phase-tuning range will be more convenient to obtain different kinds of patterns, the phase coverage less than 360° and the amplitudes fluctuating slightly in our design are not influencing factors for our DOA detection.

### 2.2 Random forest ensemble learning method

Obtaining DOA from similar transmitted wave information is an inverse problem. Indistinguishable data processing is the main obstacle to machine learning. The random forest (RF) is a type of ensemble learning and is widely used in the classification [51], [52], [53] and regression [54], [55], [56] of various data types. In comparison with the single model, ensemble learning integrates multiple models and pays more attention to the laws hidden in the data, which is appropriate for data with slightly different rules in pursuit of high precision. In this study, the DOA prediction aims at high precision and high accuracy, and thus, the RF has key advantages in gathering the output of all the decision trees to make the final prediction. Each decision tree is capable of mastering the features of different data combinations and repeatedly learning the same data features. This characteristic is potentially quite useful as it can provide a clue for examining the underlying physics behind the observed phenomena based on the importance degree of each input variable, thereby extending beyond the machine-learning-based prediction.

**Figure 3:**
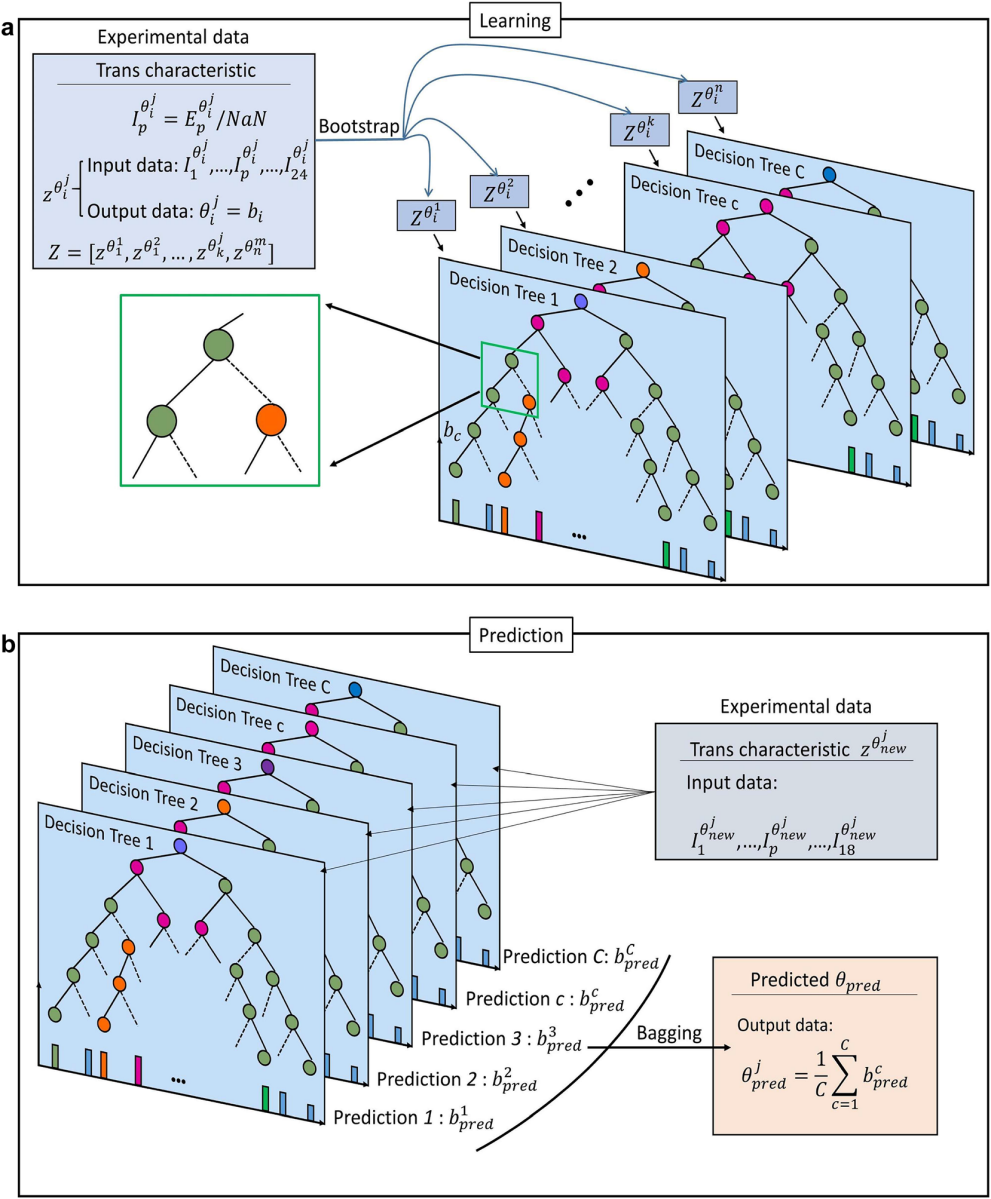
Schematic and working principle of RF. It creates many decision trees to learn the relationship between the distribution of the field intensity and the direction of the incoming waves. (a) Learning process. The experimental data are independent-trained in each model through random bootstrap sampling with put back. (b) Prediction process. Through bagging, the predicted result of the RF is defined as the average output from all decision trees.

On the other hand, the precision and the range of the elevation angle θ limit the capacity of the data set, which is more suitable for RF rather than neural network training. As shown in Figure 3(a), the RF creates multiple decision trees and generates independent training sets for each tree through random sampling with put back (bootstrap sampling). In this case, the bootstrap sampling (Figure 3(a)) shows a unique advantage in expanding a moderate number of datasets into a large volume of datasets, as they are required to improve the prediction accuracy (see Supplementary Note 5 for additional details on model learning and prediction). In our model (Figure 3(b)), a set of data should contain the transmitted wave field intensities corresponding to 24 phase patterns of the metasurface. For robustness and fault tolerance, the value of $I_{p}^{\theta_{i}^{j}}$ is either the amplitude or an artificial NaN (not a number). Therefore, the real input to the RF is a set of data containing multiple NaNs. In a trained RF, each decision tree will produce a prediction of the incident angle, $b_{pred}^{c}$ . Finally, the output of each decision tree is integrated through bootstrap aggregation, that is, the final output $b_{pred}$ is obtained by taking the average of the predicted values from all of the decision trees (a total of C decision trees).

## 3 Results and discussion

**Figure 4:**
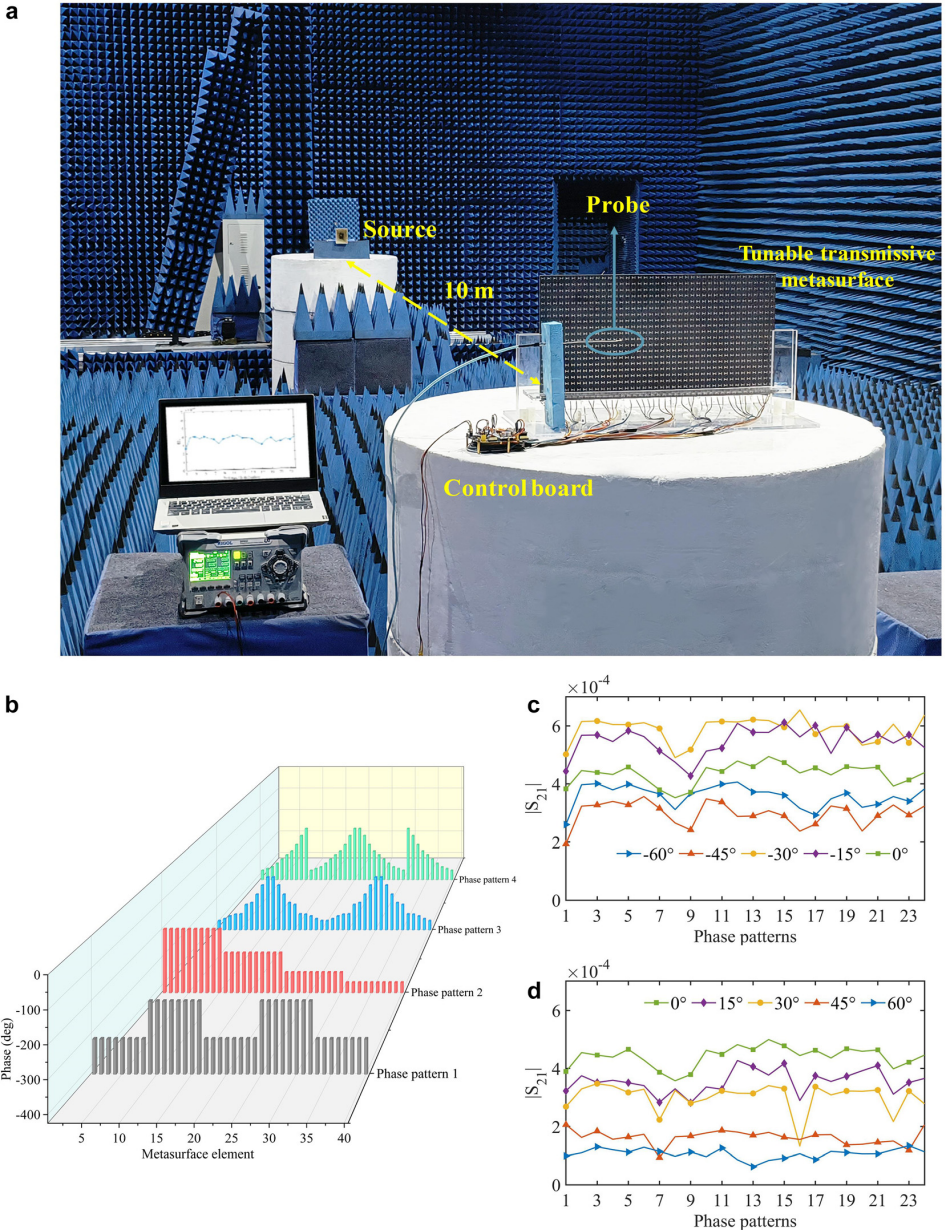
Experimental setup and field intensity distribution in specific phase patterns. (a) The TM polarized transmitting antenna is placed approximately 10 m in front of the metasurface (Supplementary Note 2). The probe is placed 4 cm behind the metasurface, and they are held in the same position relative to each other on the turntable. In this scenario, the rotation of the metasurface causes the transmitting antenna to be relatively tilted such that the beam is obliquely incident into the metasurface. When there are incoming waves, the metasurface will perform multiple phase pattern updates for manipulation. The probe receives the transmitted waves in real time and feeds the data into the RF to instantly obtain the incident angle. (b) Phase patterns for transmission wave regulation. Among them, phase patterns 1 and 2 change steeply, whereas phase patterns 3 and 4 increase or decrease gradually along the *x* direction. (c) and(d) The field intensity distribution of incoming waves from a wide range of directions after passing through the metasurface in various phase patterns. The variations of transmitted waves from the same side are similar. See additional details in Supplementary Note 6.

To verify the proposed DOA estimation method, a proof-of-concept experiment is conducted at 9.5 GHz. Here, without loss of generality, we only consider the elevation angle θ . Nevertheless, this detection strategy for the DOA is applicable for two-dimensional incoming wave detection at arbitrary frequencies. Figure 4(a) presents the entire experimental setup arranged in the same way as in Figure 1. In this system, the metasurface and the probe are relatively fixed, and they are located together on the turntable to achieve an oblique plane-wave incidence. As the incident signal, the transmitting antenna emits transverse magnetic (TM) waves from a sufficiently long distance, which can be approximated as plane waves. The horn antenna and the probe are connected to the two ports of an Agilent vector network analyzer. In the experiment, to fulfill the requirements for the differential regulation of beams with a wide range of incident angles, the selected phase patterns include abrupt and gradual phase changes along the *x* direction (Supplementary Note 3). Notably, the method proposed herein is based on the differentiated electric field (see Supplementary Note 6 for the variation of field intensities in each phase pattern) at the same location (point) to perceive the DOA; therefore, a phase distribution that can achieve a specific function (e.g., focusing and deflection) is not necessary. To obtain the direction information of the incident wave, the control board (Supplementary Note 4) delivers 24 voltage control instructions to manipulate the phase patterns (Figure 4(b)). Thus, multiple sets of transmitted wave data corresponding to each θ are acquired (Figure 4(c)). In practice, the switching speed of the varactor diode is 3 ns. The microcontroller unit clock rate is 72 MHz, and the clock frequency is 18 MHz when sending voltage control instructions. Thus, it is possible to confirm that the entire data acquisition time is almost real-time, i.e., approximately 0.11 ms for each data sampling with 24 phase patterns.

To better verify the performance of our method, the incident angle interval in the experimental data is $0.1^{{}^{\circ}}$ , and the range is $\left[-60^{{}^{\circ}},60^{{}^{\circ}}\right]$ . This way, we build up the dataset, i.e., 1201 incident angles under 24 voltage distributions; this is far from meeting the data amount requirements for neural network training. To validate the robustness of our model, we randomly select eight (a third of each set of data) groups of field strength data and artificially rewrite them as missing values (NaN). Thus, the actual input data to the RF is 24 electric-field intensity values containing the NaN values. Moreover, owing to the uncertainty of the locations of missing values, the data set corresponding to each angle expands in the manner of combination of $C_{24}^{16}$ ( $∼7.4\times 10^{5}$ ), scaling up to the order of hundreds of thousands. For angle $\theta_{i}^{j}$ , $z^{\theta_{i}^{j}}=\left\{I_{1}^{\theta_{i}^{j}},I_{2}^{\theta_{i}^{j}},\ldots ,I_{p}^{\theta_{i}^{j}},\ldots ,I_{24}^{\theta_{i}^{j}}\right\}$ , where $z^{\theta_{i}^{j}}$ is the set of data for the incident angle $\theta_{i}^{j}$ . For data preprocessing, we add the k-nearest neighbor algorithm to fill in the missing values. Then, divided into training and testing sets, the normalized and the randomly shuffled data are put into the RF to learn the relationship between the field strength and the incident angle θ . For illustrative purposes, we define the error, $Error_{abs}=\left|\theta_{predict}-\theta_{true}\right|$ , as within $0.1^{{}^{\circ}}$ , $0.5^{{}^{\circ}}$ and $1^{{}^{\circ}}$ , respectively. In addition, to reflect the prediction of the whole sample, the error mean and the variance are applied to measure the model performance with adequate test samples. Furthermore, to exemplify the model performance at small angles (less than $5^{{}^{\circ}}$ ), we measure the model accuracy in terms of $Error_{small}=\left|\theta_{predict}-\theta_{true}\right|/\theta_{true}$ .

**Figure 5:**
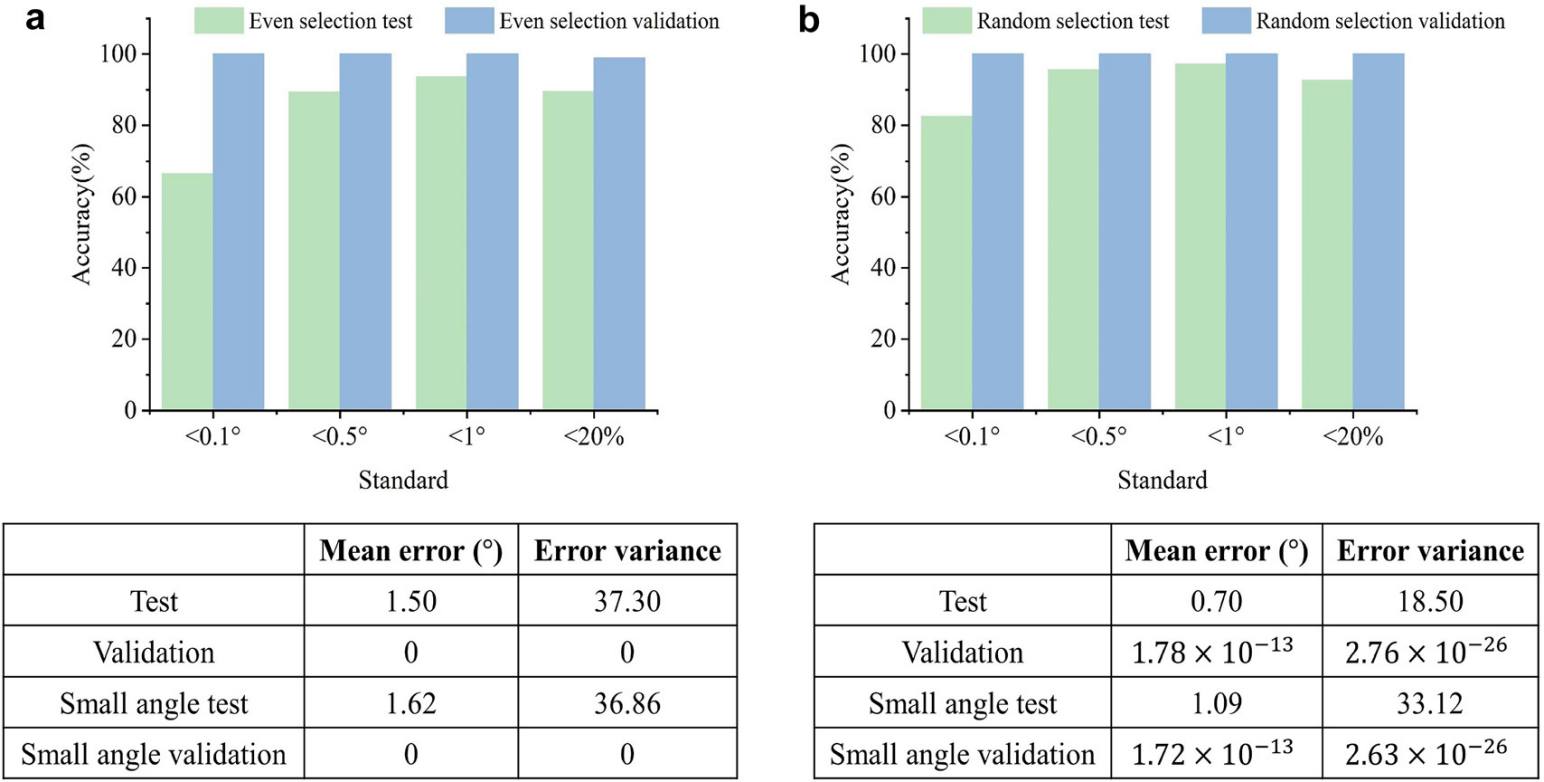
RF training results. The proportion of testing samples in ${\mathrm{Error}}_{\mathrm{abs}}\leq 0.1^{{}^{\circ}}$ , ${\mathrm{Error}}_{\mathrm{abs}}\leq 0.5^{{}^{\circ}}$ , ${\mathrm{Error}}_{\mathrm{abs}}\leq 1^{{}^{\circ}}$ , and ${\mathrm{Error}}_{\mathrm{small}}\leq 20\%$ are shown in the histogram. (a) Result of the even selection of training samples at an interval of 1°. In an even selection, the accuracy within 0.5° and 1° can both reach over 90%. Small angle predictions can also be up to 90% accurate. (b) Result of randomly selecting 30% of dataset as the training samples. When training samples are randomly selected, the accuracy of each standard is improved, and the errors of more than 95% test samples are within 0.5°.

In training, we adopt two training strategies. First, given that the distributions of the field intensities between angles are nonlinear, the angles used for training are uniformly selected at $1^{{}^{\circ}}$ intervals to learn the hidden relationship between θ and the field intensity to the maximum extent. In this case, 121 sets of angles are utilized for training, accounting for 10% of all incident angles. For each angle in the training and test sets, we randomly select 20 different NaN configurations. The NaN position distributions of the training and test sets are independent of each other. During training, we divide 30% of the data as a validation set. Figure 5(a) shows the prediction accuracies for various criteria. In the test set, approximately 90% of the test set meets $Error_{abs}<0.5^{{}^{\circ}}$ . In addition, 89.5% of the small-angle sample errors are confined to $Error_{small}<20\text{\%}$ .

In the second training method, we arbitrarily select a certain proportion of angles as the training set, each of which has 20 NaN position distributions. For a more intuitive comparison, we randomly extract 30% of the data for training with an unknown angular sampling precision. Still, the test data contain all the experimental data. Figure 5(b) shows the accuracies for different accuracy standards. The mean error of the test set is $0.7^{{}^{\circ}}$ . In $Error_{abs}<0.5^{{}^{\circ}}$ and $Error_{abs}<1^{{}^{\circ}}$ , the accuracies are above 95%, 95.6%, and 97.2%, indicating that the model has extraordinary performance in detecting the direction of the incoming wave. For $\theta_{true}<5^{{}^{\circ}}$ , 92.6% of the angles are perfectly predicted, proving that the model is also suitable for the prediction of small angles. For one-dimensional angle detection, the proposed method exhibits excellent performance, within a $0.5^{{}^{\circ}}$ prediction error. Additional details regarding the model performance can be found in Supplementary Note 7. The average training times of both methods are 2.7 s and 14.8 s, respectively. The training processes are trained using Python version 3.7.0 and Scikit-learn machine learning library on a server (GeForce GTX 1650 GPU and Intel(R) Core(TM) i7-9700 CPU @ 3.00GHz with 32GB RAM).

The intelligent metasurface-assisted approach to estimate DOA presented herein shows its flexibility in the arbitrary selection of training data and robustness in the absence of field strength data. For a clearer analysis and explanation, we adopted two training methods, i.e., uniform selection and random selection for training data, as evaluated by five indicators. According to both methods, more than 90% of the angle prediction errors are less than $1^{{}^{\circ}}$ , and more than 85% are within $0.5^{{}^{\circ}}$ , showing an excellent angular resolution. It can be concluded that when the training set is sufficiently large, detection with an excellent angular resolution of $0.1^{{}^{\circ}}$ and extremely high accuracy can be achieved. Notably, in practical applications, the probe can be altered with an independent high-frequency processing module, and the data can be synchronously postprocessed, forming a fast and real-time complete detection system.

## 4 Conclusions

In conclusion, we experimentally demonstrate a machine-learning-enabled metasurface for DOA estimation that relaxes the heavy reliance on complicated antenna arrays and high-cost computers; moreover, it performs real-time detection. Owing to the well-trained machine-learning model, the intelligent estimation method is exempt from the heavy burden on the computational hardware in conventional DOA determinations. The measured results show that with sufficient training samples, an extremely high accuracy can be achieved. Wide-band and two-dimensional (azimuth angle and elevation angle) DOA detection can also be achieved by rationally designing the desired meta-atom. Moreover, cooperating with more sophisticated network configurations, we visualize our work could be a powerful strategy for the development of intelligent DOA detection.

## Supplementary Material

Supplementary Material
