# Supplementary material for: Machine–learning-enabled metasurface for direction of arrival estimation
Source: Nanophotonics. 2022 Jan 11;11(9):2001–10. doi: 10.1515/nanoph-2021-0663 (PMC11501464; doi:10.1515/nanoph-2021-0663)
Supplement: Supplementary file 1 — Supplementary Material [file j_nanoph-2021-0663_suppl.docx]

**Supplementary Information for**

**Machine Learning Enabled Metasurface for DOA Estimation**

*Min Huang^1,2^, Bin Zheng^1,2^*, Tong Cai^1,2,3^*, Xiaofeng Li^3^, Jian Liu^1^, Chao Qian^1,2^ and Hongsheng Chen^1,2^**

^1^Interdisciplinary Center for Quantum Information, State Key Laboratory of Modern Optical Instrumentation, ZJU-Hangzhou Global Scientific and Technological Innovation Center, Zhejiang University, Hangzhou, 310027, China.

^2^International Joint Innovation Center, Key Lab. of Advanced Micro/Nano Electronic Devices & Smart Systems of Zhejiang, The Electromagnetics Academy at Zhejiang University, Zhejiang University, Haining, 314400, China.

^3^Air and Missile Defense College, Air Force Engineering University, Xi’an, 710051, China

*Corresponding author: zhengbin@zju.edu.cn (B. Zheng), caitong326@zju.edu.cn (T. Cai), hansomchen@zju.edu.cn (H. Chen)

**Supplementary Note 1:** **The meta-atom design of metasurface**

As shown in Supplementary Fig. S1, the period is 15mm (4.75$\lambda$). Each meta-atom is composed of four metal layers separated by three substrate layers and bonded by two films. The metal layers from top to bottom are radiating layer, ground layer, bias layer and receiving layer. The radiating and receiving layer are almost the same, both H-shaped slots, except the varactor diode position. The ground layer is a cross with high transmission efficiency that extends to the edge, reducing the number of extra lines and joining the ground of all the units together. The bias layer consists of two crescence-shaped metal plates and a metal strip with the same length as the period to connect all the units in the y direction without additional cable power needed. The top and bottom layers of the H-shaped horizontal bar are equipped with a varactor diode (SMV2019-079LF), but placed in the opposite direction. The two ends of two varactor diodes are connected to the ground layer and the bias layer through asymmetric metallized via-holes on the same horizontal line. The three substrate layers are all composed of F4B with a dielectric constant of 3.5 and a loss tangent of 0.003 while the thickness is 0.5mm for the substrate layer that separates the ground layer from the bias layer and 2mm for the other two substrate layers. The detailed size information is shown in Table 1.

**
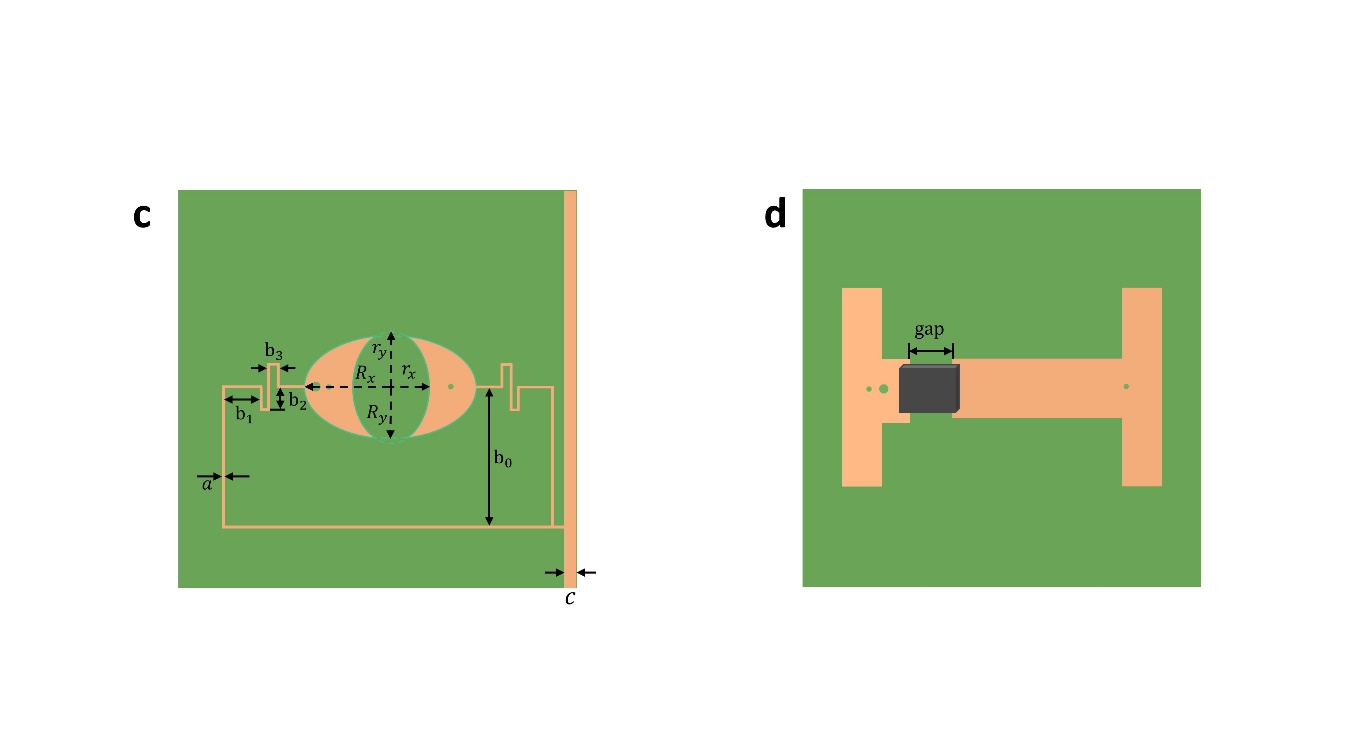
**

**
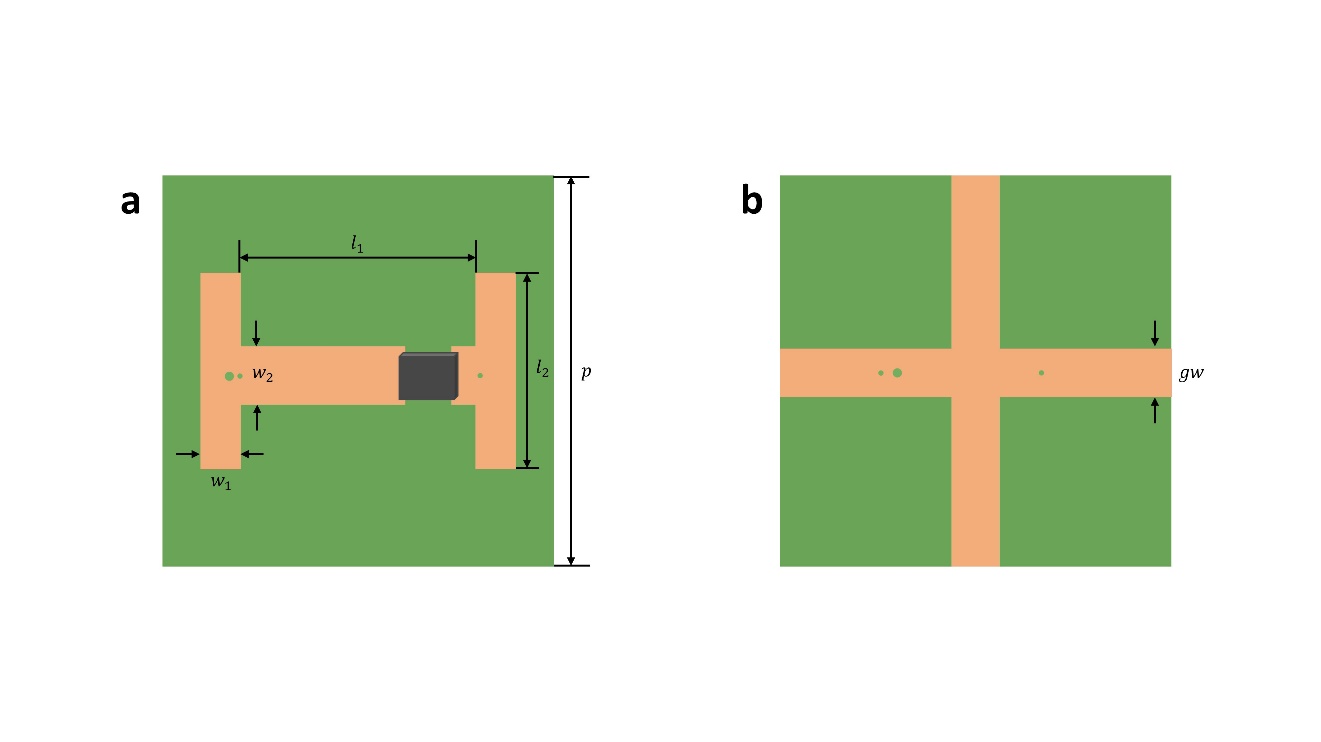
**


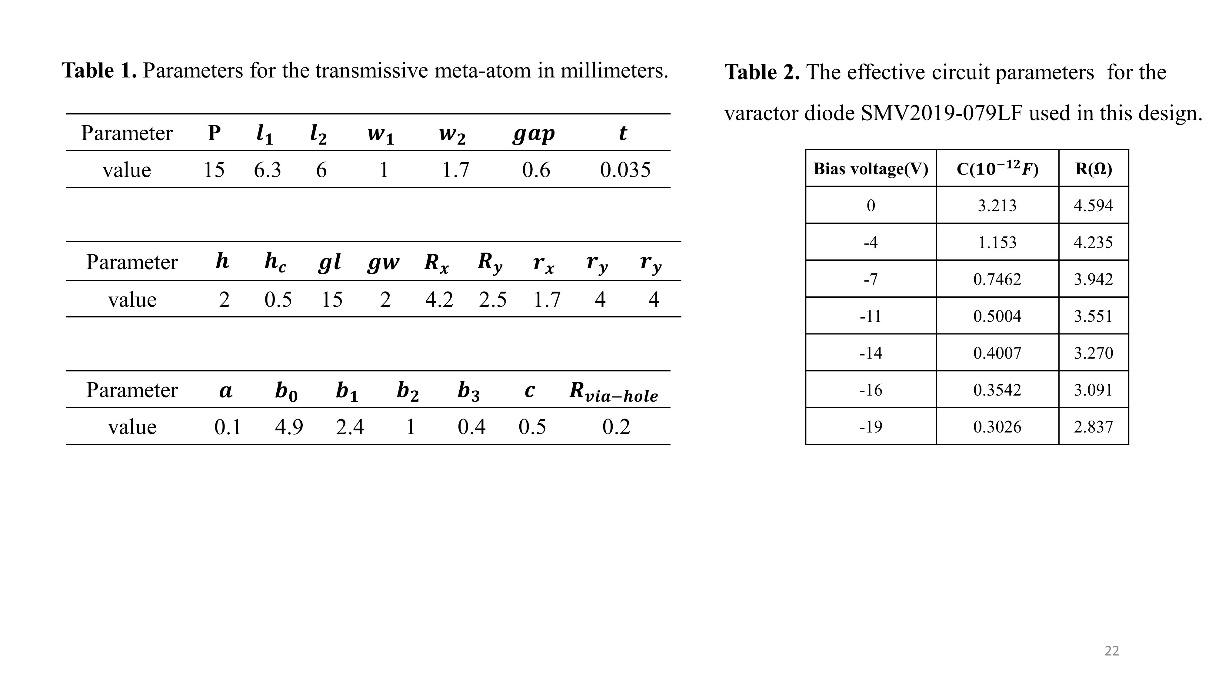


**Fig. S1:** Detailed dimensional specification of the designed meta-atom. (**a**) The top layer. (**b**) The ground layer. (**c**) The bias layer. (**d**) The bottom layer.

**Supplementary Note 2:** **The fabrication of metasurface sample**

A total of 800 units make up the metasurface ($600mm\times300mm$), 40 in the x direction and 20 in the y direction. The tunable metasurface is integrated with 1600 varactor diodes with two varactor diodes per unit, which can be controlled independently by control board (Supplementary Note 4). Considering the interference with the transmission of wave, the power supply of the metasurface is designed at the bottom. For the sake of more firm support for the metasurface without affecting the propagation of electromagnetic waves, a base consisting of acrylic sheets is employed to hold the metasurface and place it on the turntable.

**Supplementary Note 3:** **The elaboration of phase pattern for DOA estimation and field intensity distribution**

Each unit of the metasurface is separately regulated in the x direction and in the y direction, so the phase distribution is one-dimensional. We detect the direction of incoming wave according to the amplitude difference of transmitted wave and do not require the realization of specific functional phase distribution. The phase distribution in the experiment is 24 randomly selected phase distribution.


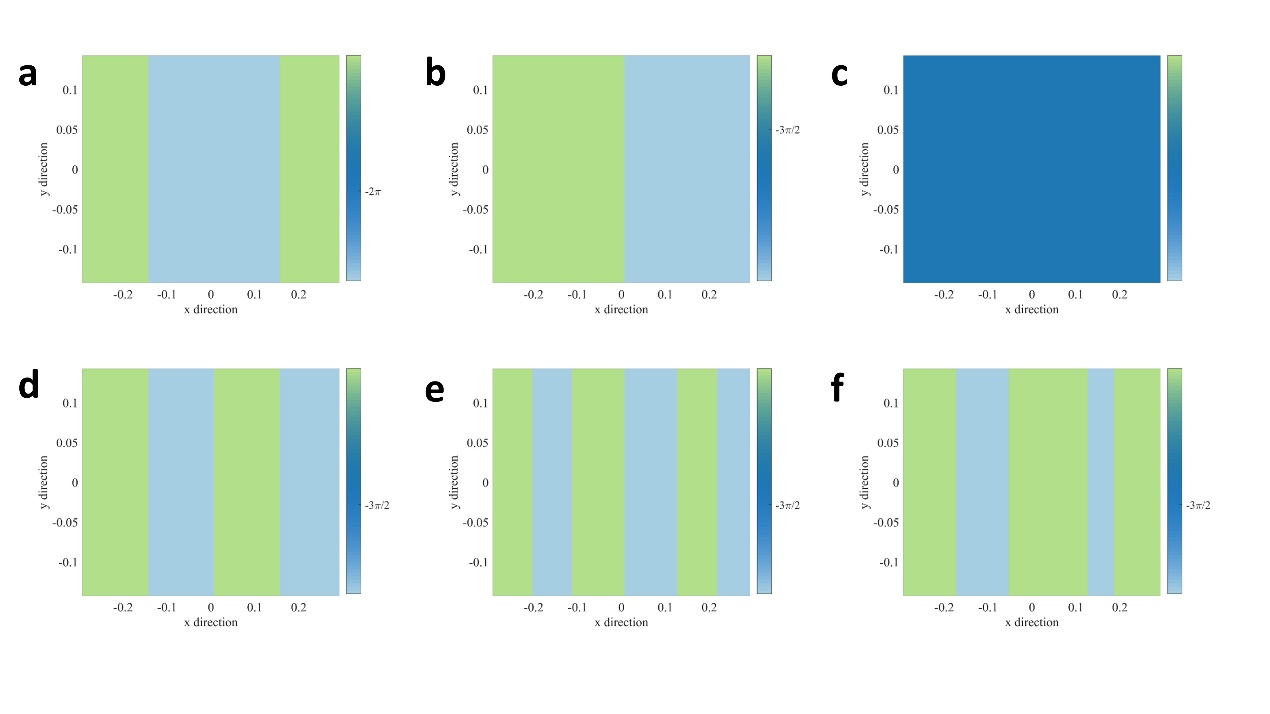


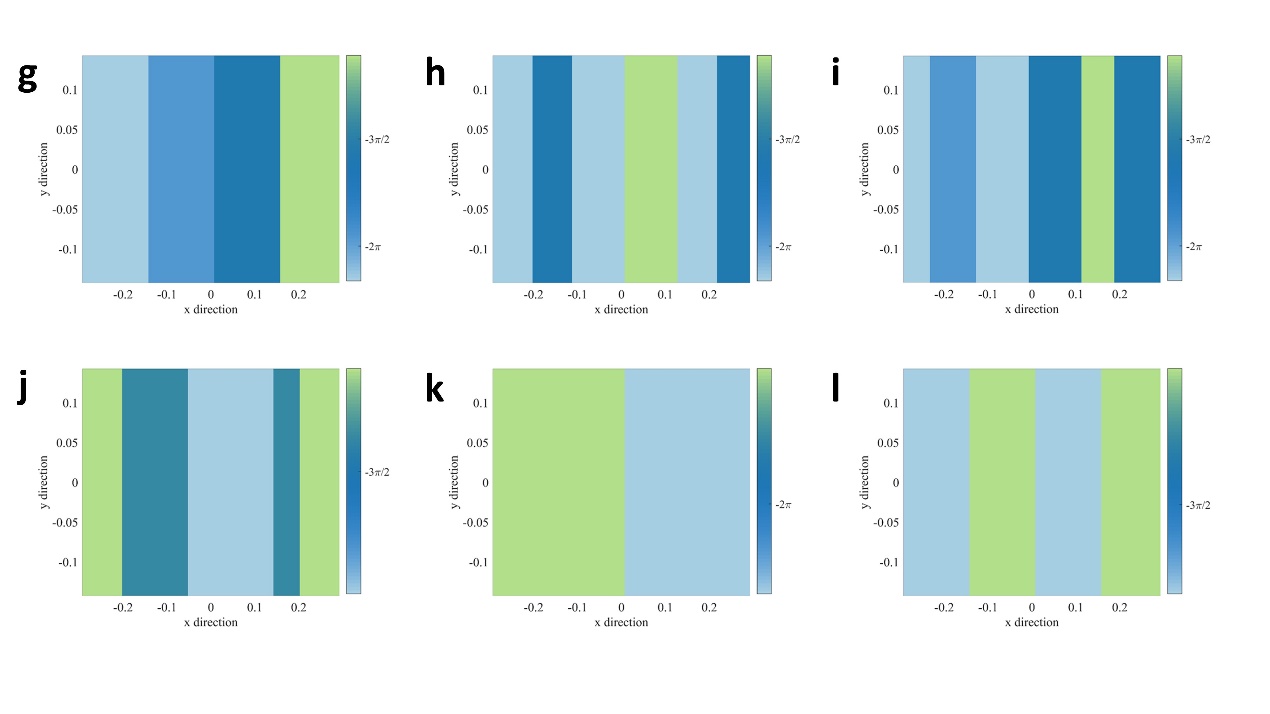


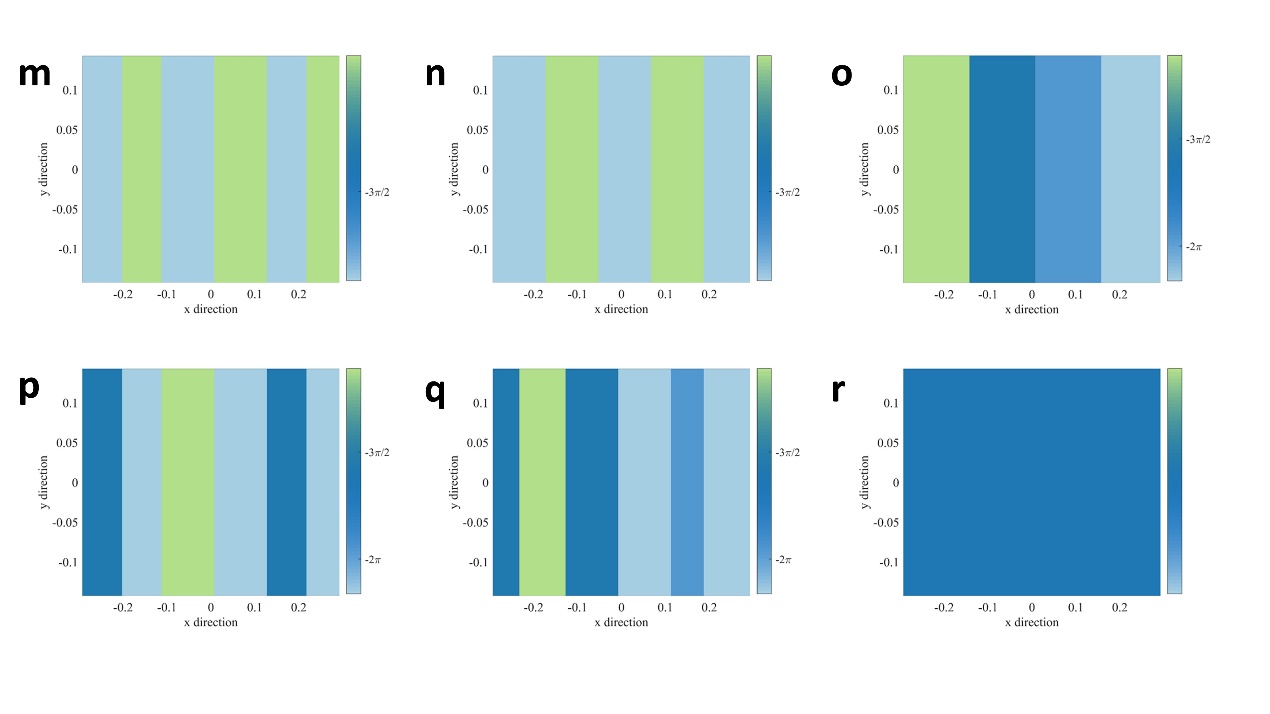


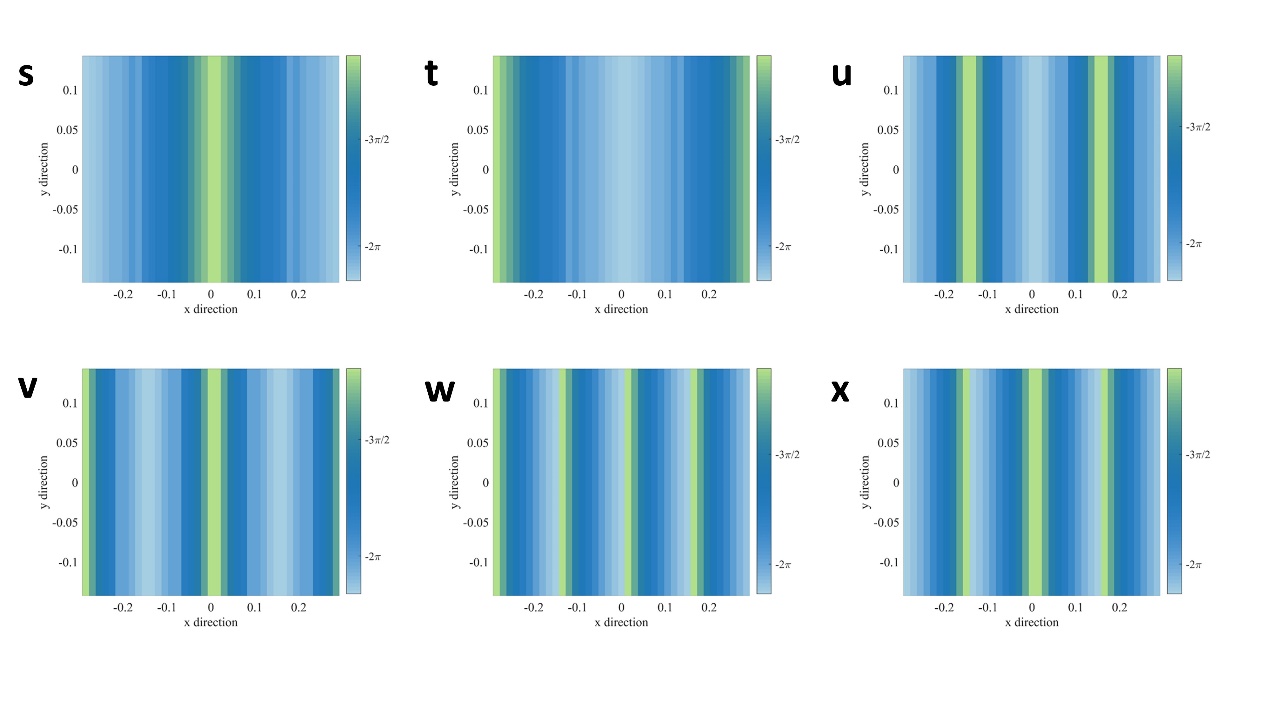


**Fig. S2:** Phase patterns in the experiment. (**a-r**) The phase changes sharply and the elements within a certain range remain unchanged. (**s-x**) In the x direction, the phase increases or decreases gradually.

**Supplementary Note 4:** **Voltage control module and MCU part**

The working voltage of the varactor diodes is from 0 to 19 volts. We use STM32 as a microprocessor, outputting instructions to the digital-to-analog converter (DAC) to output the desired voltage. DAC8718, a low-power, octal, 16-bit and serial-input DAC chip, can output a unipolar 0V to +30V voltage when operating from a +30.5V (or higher) power supply. STM32 communicates with DAC8718 through the serial SPI protocol, which belongs to one-to-many control and simplifies the complexity of hardware. We integrate 8 pieces of DAC8718 on the voltage control board, each piece can output 8 channels of voltage independently, so the control board has 64 channels of independent voltage output IO port. The voltage control board uses 24V DC power supply, and supplies power to DAC8718 and STM32 through the 5V step-down module. The output of the 32V step-up module is used as the reference voltage of DAC8718. The DAC8718 operates at a frequency of up to 50 MHz, promising extremely small voltage switching cycles. The clock frequency of the STM32 is 72MHz, which guarantees excellent running speed.


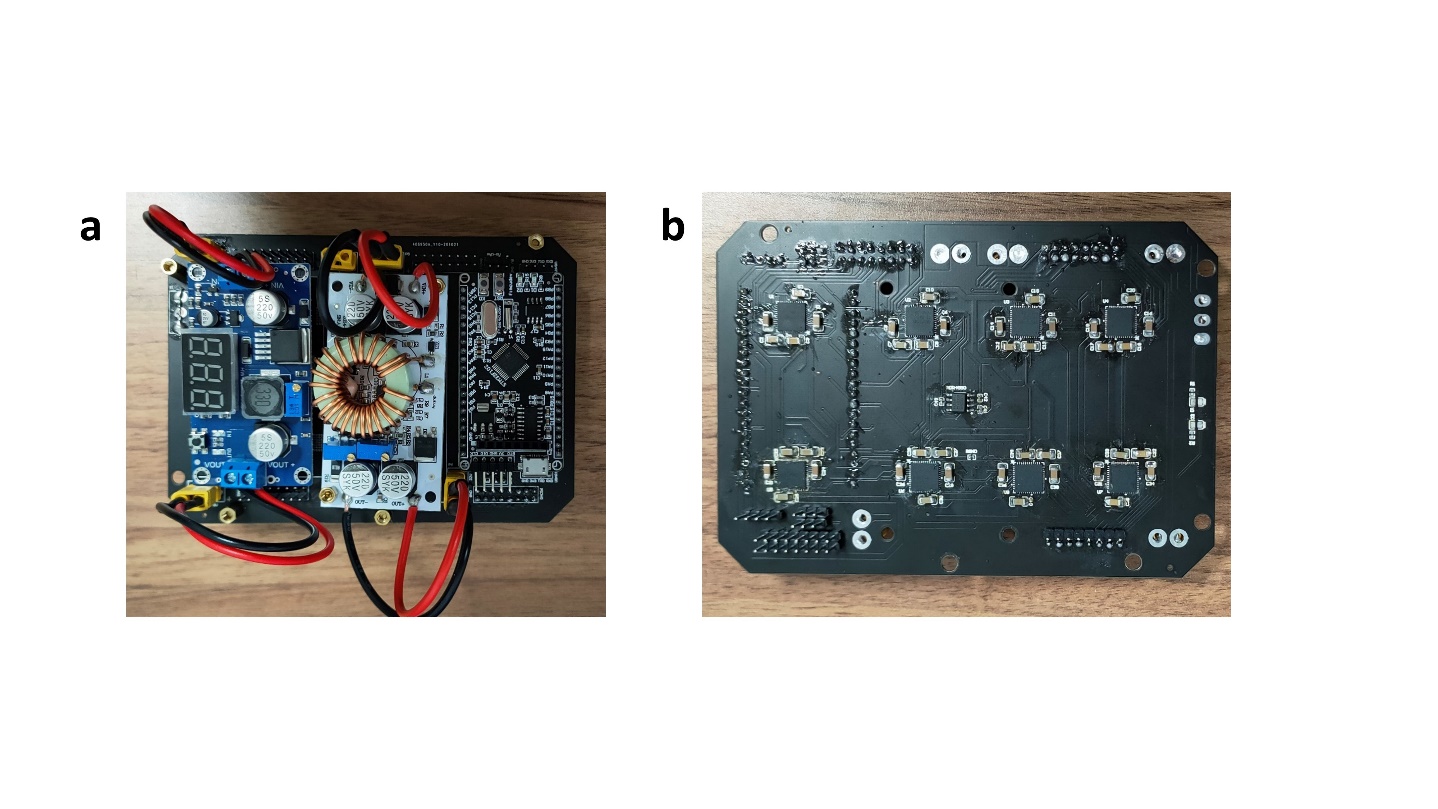


**Fig. S3:** Front and back sides of the control board. Placing the chip and module on both sides not only saves space, but also enhances heat dissipation and improves performance. (**a**) The front of the control board contains an MCU(STM32), a 24V step-down 5V module and a 24V step-up 32V booster module. (**b**) There are 8 DAC8718 in the back as bias voltage output.

**Supplementary Note 5**: **Random forest ensemble-learning method**

In learning process, under the bootstrap sampling principle, RF will create $C$ new groups of training data randomly selected from $Z$ with replication allowed for independent learning of $C$ decision trees. For example, $Z^{1}=\{z^{\theta_{1}^{1}},z^{\theta_{3}^{2}},\ldots,z^{\theta_{2}^{2}},\ldots,z^{\theta_{k}^{2}}\}$, $Z^{2}=\{z^{\theta_{n}^{l}},z^{\theta_{5}^{6}},\ldots,z^{\theta_{6}^{6}},\ldots,z^{\theta_{5}^{6}}\}$, where $k$ and $l$ are integers $(k\leq n, l\leq m)$ and some data sets are repeatable (e.g., $z^{\theta_{5}^{6}}$ appears twice in $Z^{2}$) while some does not exist, such that increases the diversity of the data set. The training data will traverse from the first node of the decision tree to the last leaf node, and there will be an output after each leaf node, $b_{c}$. Each set of data put into our model contains the transmitted wave field intensities corresponding to the 24 phase patterns of metasurface. That means, each $\theta_{i}^{j}$ owns 24 different amplitude data, $z^{\theta_{i}^{j}}=\{I_{1}^{\theta_{i}^{j}},I_{2}^{\theta_{i}^{j}},\ldots,I_{p}^{\theta_{i}^{j}},\ldots,I_{24}^{\theta_{i}^{j}}\}\to\left\{ \theta_{i}^{j} \right\}$, where $p$ indicates the phase pattern and $j$means $j$th data item of $\theta_{i}^{j}$. The value of $I_{p}^{\theta_{i}^{j}}$ can be artificially set to NaN. One third (8/24) of the values in a set of data will be given NaN. The locations of NaNs determine the difference in data. For example, for angle $\theta_{i}^{j}$,$z^{\theta_{i}^{j}}=\{I_{1}^{\theta_{i}^{j}},I_{2}^{\theta_{i}^{j}},\ldots,I_{p}^{\theta_{i}^{j}},\ldots,I_{24}^{\theta_{i}^{j}}\}$, where $I_{1}^{\theta_{i}^{j}},I_{6}^{\theta_{i}^{j}},I_{7}^{\theta_{i}^{j}},I_{8}^{\theta_{i}^{j}},I_{9}^{\theta_{i}^{j}},I_{15}^{\theta_{i}^{j}},I_{18}^{\theta_{i}^{j}},I_{24}^{\theta_{i}^{j}}=NaN$and $I_{2}^{\theta_{i}^{j}},I_{6}^{\theta_{i}^{j}},I_{7}^{\theta_{i}^{j}},I_{8}^{\theta_{i}^{j}},I_{9}^{\theta_{i}^{j}},I_{15}^{\theta_{i}^{j}},I_{18}^{\theta_{i}^{j}},I_{24}^{\theta_{i}^{j}}=NaN$ are two different sets of data, although they differ by only one element (the first one). Thus, for angle $\theta_{i}^{j}$, the input data that can represent it is changed from one set to two sets, so the data is also expanded. $Z=\{z^{\theta_{1}^{1}},\ldots,z^{\theta_{i}^{j}},\ldots{,z}^{\theta_{n}^{m}}\}$ is the collection of all data for machine learning.

And in the prediction, RF will generate data sets according to the number of decision trees, so that each decision tree will generate a predicted value. And the final output $b_{pred}$ is the average of all the predicted values.

**Supplementary Note 6:** **The variation of transmitted wave intensities in phase patterns**

Under the 24 phase patterns, the variation trend of transmitted wave field intensity is consistent with the incident angle from ${-60}^{^{\circ}}$ to ${+60}^{^{\circ}}$.


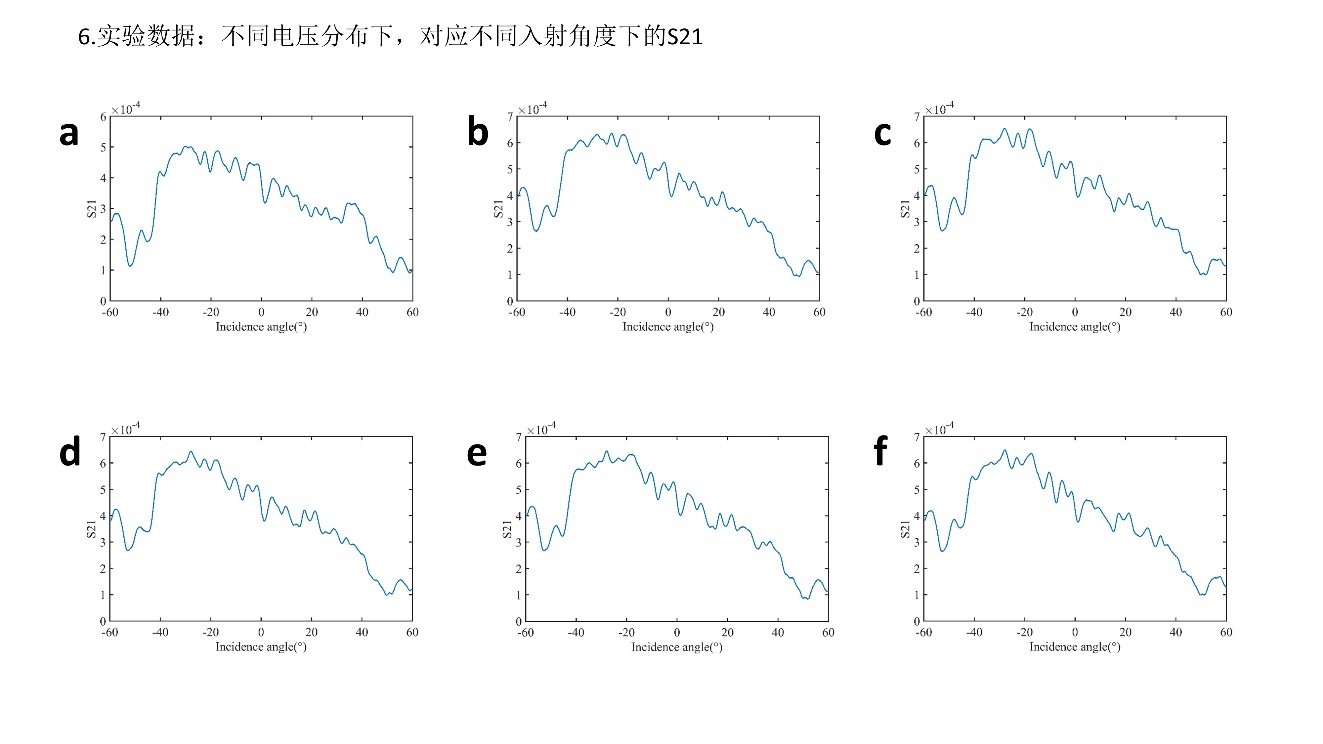


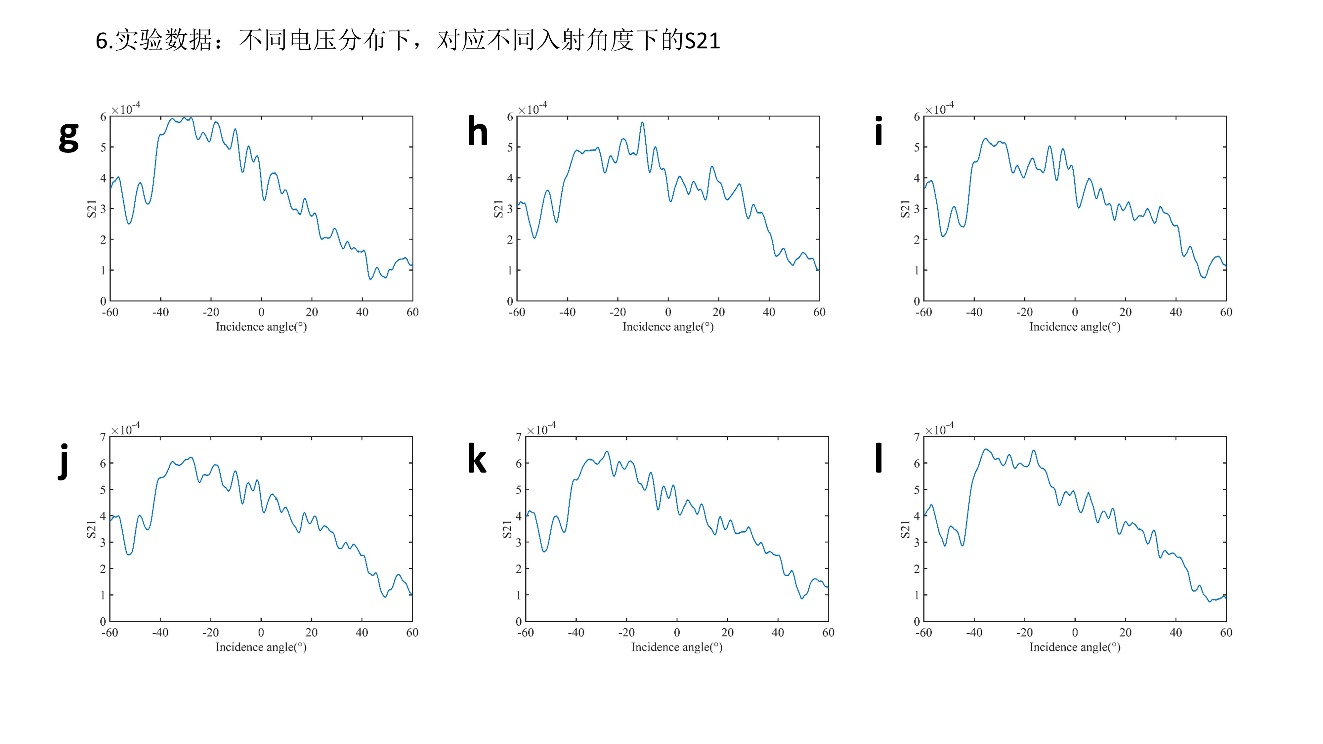


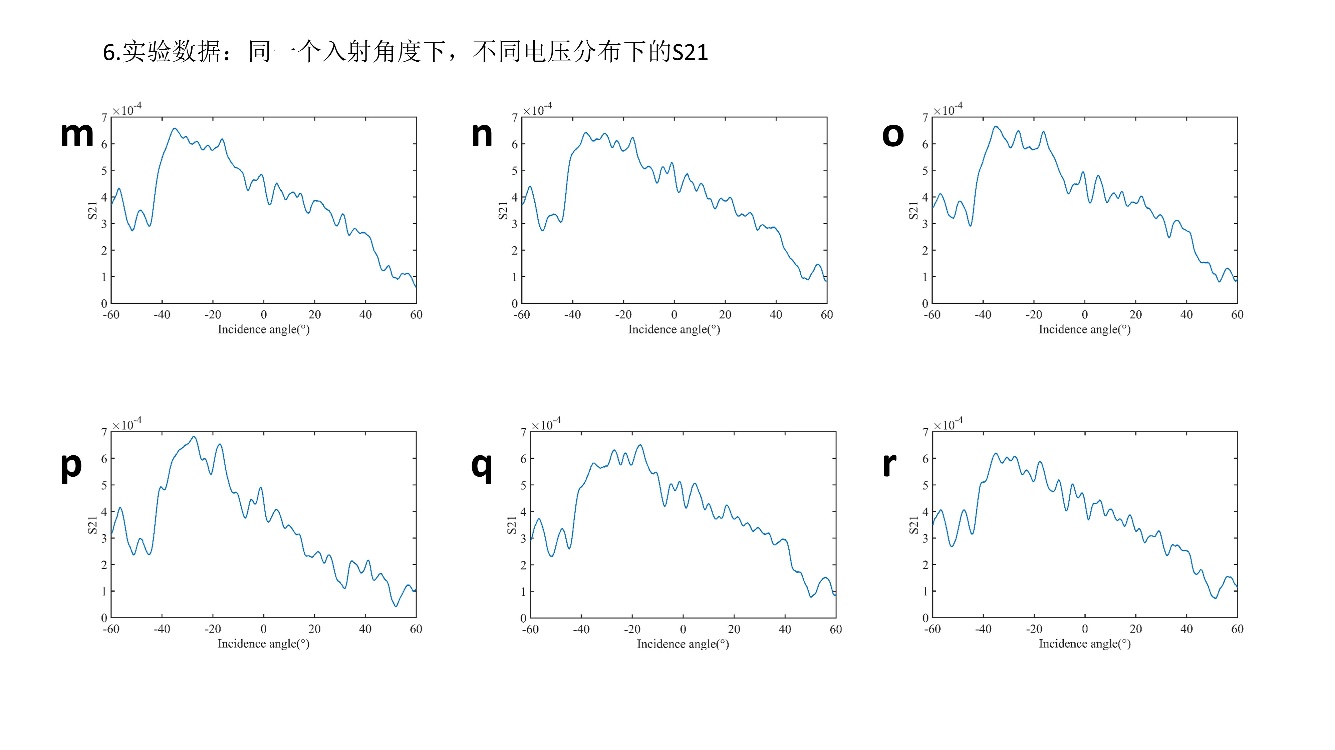


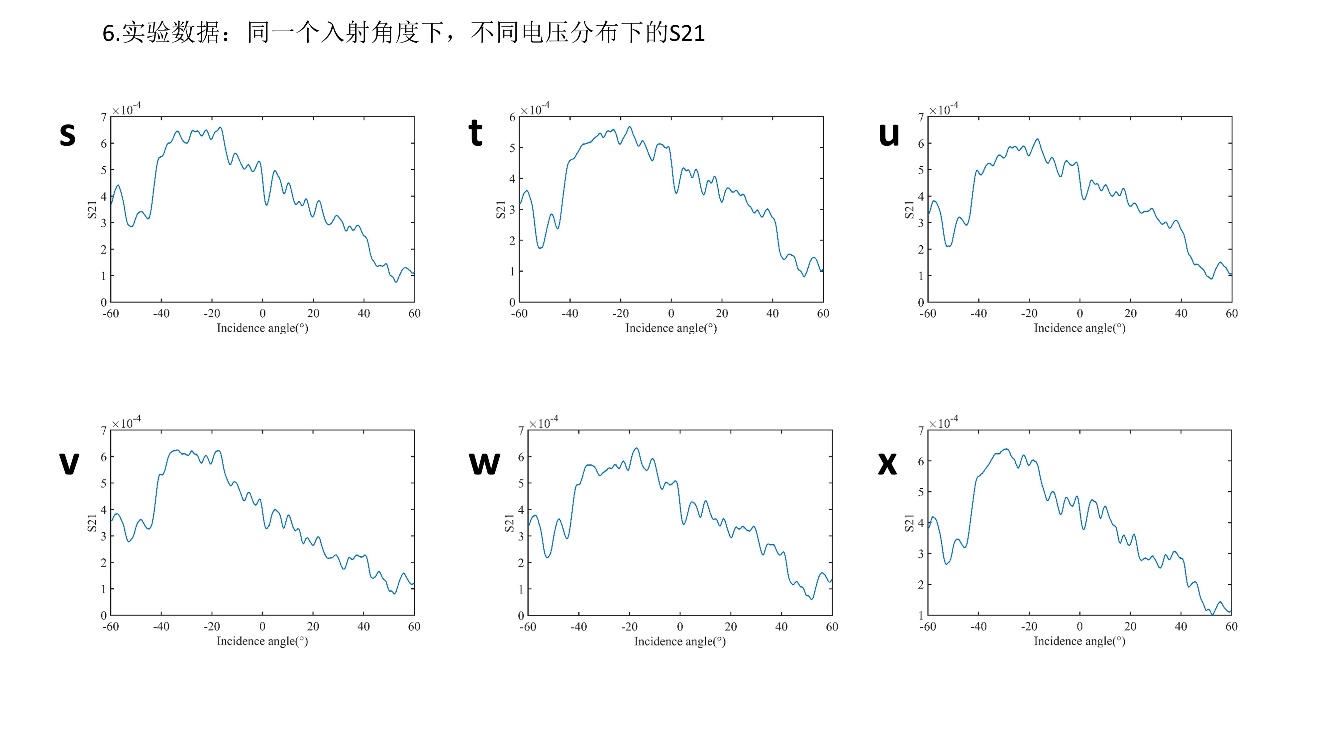


**Fig. S4:** Variation of transmitted wave field intensity in different patterns

**Supplementary Note 7: The discussion on the performance of random forest method**

There are two training strategies. The difference between them is whether the training samples are taken randomly. The first one is even selection. In the actual construction of the training set, field intensity data under 24 phase patterns are input, among which the positions of 8 sets of NaN are sampled at random. 20 different NaN position distributions are chosen for each angle in the training set. The final training set size is $2420 (121\times20)$ pieces of data, corresponding to 121 angles. To evaluate the performance of universality and reliability of detection as thoroughly as possible, all the experiment angles data will be applied to testing too. Similarly, each $\theta$ is in correspondence with 20 NaN position distributions by random selection, so the test set is made up of $24,020(1201\times20)$ pieces of data. The NaN position distributions of training set and test set are independent of each other. During the training, we divide 30% of the data as a validation set, and the NaN distributions of the validation set are different from those in the training set. Compared to ${Error}_{abs}<{0.5}^{^{\circ}}$, the accuracy only rises to 93% in ${Error}_{abs}<1^{^{\circ}}$, implying that most of the prediction error is confined to ${0.5}^{^{\circ}}$.

In the random selection training, the training set consists of randomly selected data in a certain proportion. In ${Error}_{abs}<{0.1}^{^{\circ}}$, compared with the previous training method, the accuracy of the test set increases from 66.5% to 82.5%. For the other two criteria, accuracy is more than 95%, which is the embodiment of extremely high accuracy.
